# Cardiac Autonomic Function in Patients with Systemic Sclerosis: The Impact of Exercise Training and Detraining

**DOI:** 10.3390/sports13080267

**Published:** 2025-08-13

**Authors:** Maria Anifanti, Andriana Teloudi, Alexandros Mitropoulos, Niki Syrakou, Eleni Pagkopoulou, Eva Triantafyllidou, Carina Boström, Louise Pyndt Diederichsen, Tiziana Nava, Theodoros Dimitroulas, Markos Klonizakis, Evangelia Kouidi

**Affiliations:** 1 Laboratory of Sports Medicine, Department of Physical Education and Sports Science, Aristotle University of Thessaloniki, 57001 Thessaloniki, Greece; manyfant@phed.auth.gr (M.A.); teloudia@gmail.com (A.T.); nikisir5@gmail.com (N.S.); 2 Lifestyle, Exercise and Nutrition Improvement (LENI) Research Group, Department of Nursing and Midwifery, Sheffield Hallam University, Sheffield S10 2BP, UK; alexandros.mitropoulos@shu.ac.uk (A.M.); m.klonizakis@shu.ac.uk (M.K.); 3 4th Department of Internal Medicine, School of Medicine, Hippokration Hospital, Aristotle University of Thessaloniki, 54642 Thessaloniki, Greece; elenipag4684@gmail.com (E.P.); evatrian@gmail.com (E.T.); dimitroul@hotmail.com (T.D.); 4 Department of Neurobiology, Care Sciences and Society, Karolinska Institutet, SE-104 35 Stockholm, Sweden; carina.bostrom@ki.se; 5 Department of Rheumatology, Copenhagen University, Rigshospitalet, DK-1165 Copenhagen, Denmark; louise.diederichsen@regionh.dk; 6 Department of Physiotherapy, Milan University, 20122 Milan, Italy; tiziananava.job@outlook.it

**Keywords:** systemic sclerosis, exercise training, cardiac autonomic function, cardiorespiratory efficiency

## Abstract

Adverse cardiovascular events and increased mortality are associated with cardiac autonomic nervous system dysfunction in the early stages of the systemic sclerosis (SSc), even prior to the development of cardiac fibrosis. The objective of the study was to evaluate the impact of a three-month exercise training regimen and a subsequent comparable period of detraining on the activity of the cardiac autonomic nervous system in patients with SSc. A total of forty patients with SSc were randomized to either the control group (Group COΝ) or the exercise training group (Group ET). Cardiopulmonary exercise testing was performed at baseline, three months later, and six months later to assess peak oxygen uptake (VO_2_peak). They also had 24 h electrocardiogram monitoring for heart rate variability (HRV) and heart rate turbulence analysis. The following time-domain indices were evaluated in the context of HRV analysis: the standard deviation of NN intervals (SDNN), the root mean square of successive RR interval differences (rMSSD), and the percentage of successive RR intervals that differ by more than 50 ms (pNN50). Additionally, regarding the frequency-domain indicators, the low-frequency (LF) and high-frequency (HF) components, as well as the LF/HF ratio, were evaluated. Independent *t*-tests and Chi-square tests were used for baseline comparisons, while two-way repeated measures ANOVA with Bonferroni post hoc tests assessed changes over time and between groups. Linear and multiple regression analyses were conducted to explore relationships among variables and identify predictors of HRV indices and VO_2_peak. Group ET implemented a three-month mixed-type exercise training program, while Group COΝ received standard care. Group ET improved indices of vagal activity [rMSSD by 32.6% (*p* = 0.017), pNN50 by 57.1% (*p* = 0.01) and HF by 20.1% (*p* = 0.01)] and sympathovagal activity [SDNN by 15.5% (*p* = 0.002) and LF/HF by 12.03% (*p* = 0.004)] after three months. Exercising patients also increased their VO_2_peak by 20.8% (*p* = 0.001). A robust positive correlation was observed between ΔVO_2_peak and ΔSDNN (r = 0.754, *p* < 0.001). After three months, there was no statistically significant difference in the VO_2_peak or any HRV index in the group COΝ. Compared to the baseline values, there was no statistically significant difference in group ET at 6 months, whereas the control group exhibited a decline. In summary, a three-month mixed-type exercise training program can enhance the cardiorespiratory efficiency and cardiac autonomic nervous system function of patients with SSc, as well as alleviate the deterioration that arises following the detraining period.

## 1. Introduction

Fibrosis, vascular damage, and immune disturbances are the hallmarks of systemic sclerosis (SSc), a systemic connective tissue disease that impacts internal organs to varying degrees. The complex underlying pathophysiology of the disease leads to generalized inflammation, vascular dysfunction, and fibrosis resulting from chronic ischemia, which can affect multiple organs and systems, including the skin, gastrointestinal tract, heart, and lungs [1,2]. The involvement of the heart and lungs is the main cause of mortality [3], since cardiac involvement is estimated to cause high five-year mortality rates of up to 70% [4].

The role of SSc patients’ cardiac autonomic nervous function has been studied since the early 1980s [5]. Dysfunction of the cardiac autonomic nervous system is now known to appear early in the course of the disease, even before cardiac fibrosis occurs [6,7]. The presence of cardiac autonomic nervous system dysfunction is considered an early indicator of disease progression [6,7].

The primary pathophysiological mechanism is believed to be linked to abnormal vascular reactivity and impaired microcirculation, which are the result of autonomic nervous system dysfunction [8]. Additionally, elevated pulmonary artery pressures are linked to cardiac autonomic dysfunction, even in the absence of a diagnosis of pulmonary hypertension, suggesting a preclinical dysfunction of the cardiopulmonary circulation in patients with systemic sclerosis [9]. The literature also suggests a correlation between cardiac autonomic dysfunction and cardiac function in these patients, which leads to arrhythmias [10] and changes in cardiac remodeling and function [11]. Even in patients who do not have obvious cardiac involvement, resting autonomic dysfunction also seems to be linked to decreased exercise tolerance [8].

The diagnostic evaluation of autonomic dysfunction can be performed using various tests, such as the Ewing test, which was the first method developed to assess autonomic function [12]. In contrast, heart rate variability (HRV) and heart rate turbulence (HRT) are dependable non-invasive methods for evaluating the activity of the cardiac autonomic nervous system and are well-established tools for the diagnosis and evaluation of cardiac autonomic neuropathy [13,14,15]. Adverse cardiovascular events and increased mortality in patients with SSc are reported to be associated with reduced parasympathetic and enhanced sympathetic activity, as well as reduced interaction between the sympathetic and parasympathetic nervous systems [10,16].

The management of the disturbances caused by cardiac autonomic nervous system dysfunction in systemic sclerosis is not sufficiently effective with pharmacological strategies. Nevertheless, cardiovascular and cardiac autonomic nervous systems significantly benefit from consistent exercise [17]. Studies conducted on athletes, the elderly, patients with chronic cardiovascular diseases, diabetes mellitus, chronic kidney disease, and breast cancer survivors have demonstrated that systemic exercise training can enhance the function of the cardiac autonomic nervous system [17,18,19,20,21,22]. However, the impact of exercise training on the activity of the cardiac autonomic nervous system in patients with SSc has not yet been investigated, which is indispensable for the purpose of guaranteeing exercise safety, optimizing exercise training programs, averting potential cardiac problems, and addressing critical knowledge gaps that are prerequisites for evidence-based clinical guidance. Moreover, there is a lack of research on the influence of detraining on cardiac autonomic nervous system function, which is essential for the identification of patient-specific risks, the prevention of cardiovascular health deterioration, the development of effective strategies to maintain benefits during periods without exercise and the comprehension of the duration and reversibility of autonomic changes. In young, high-level endurance athletes, a four-week detraining period resulted in a decrease in resting HRV indices and endurance performance [23]. Similarly, breast cancer survivors experienced a complete loss of all cardiac autonomic function benefits following a six-week detraining period [24].

Therefore, the purpose of this study was to investigate how cardiac autonomic nervous system activity was affected in patients with SSc after a three-month exercise training program and a similar period of detraining.

## 2. Materials and Methods

### 2.1. Participants

The study issued an open call for patients with SSc to enroll. SSc was diagnosed using the criteria established by the American College of Rheumatology and the European League Against Rheumatism [25]. Adults with SSc who had been receiving stable medical and pharmaceutical therapy for a minimum of three months were eligible to participate in the study. The presence of active digital ulcers, pulmonary hypertension, heart failure with preserved or reduced ejection fraction, atrial fibrillation, coronary artery disease, diabetes mellitus, moderate-severe valvular heart disease, liver and kidney failure, hypertension, cerebrovascular diseases, thyroid diseases or any musculoskeletal, neurological or psychological disorder that could potentially impact participation in an exercise intervention were all considered exclusionary.

### 2.2. Sample Size Estimation

The necessary sample size was determined for a two-way repeated-measures ANOVA (within-between interaction) using G*Power 3.1.9.4. The analysis necessitated a total sample of 28 patients (14 in each group) to achieve a power of 80%, with a significance level of *p* < 0.05 and an effect size f of 0.25. To guarantee an adequate final sample size for analysis, a total of 36 patients had to be recruited, assuming a 20% anticipated dropout rate.

### 2.3. Study Design

This was a randomized controlled trial. At baseline, all volunteers provided their medication and the results of their latest examinations. Those who met the inclusion criteria provided written informed consent after detailed information about the study design, objective, and methods. To mitigate external influences on the measurements, the evaluations were conducted in a controlled clinical setting with standardized environmental conditions. They underwent a 12-lead electrocardiogram (ECG), 24 h ECG monitoring, and an arm crank cardiopulmonary exercise test (CPET). Patients were instructed not to consume any alcohol or caffeine for 24 h before the assessments, which were conducted in the morning. To preserve the quality of the data and alleviate patients’ fatigue, a maximum of two patients were evaluated per day during each evaluation session, which lasted approximately 2 h. To enhance reliability, standardized rest periods were implemented. Following baseline assessment, all participants were randomized into either the exercise (group ET) or control group (group CON) using www.randomizer.org (accessed on 23 September 2022). Participants were assessed at baseline and months 3 and 6. Group CON was instructed to abstain from any structured exercise program for the six months and to follow their usual lifestyle, while group ET was given the autonomy to choose their daily schedule after the end of the 3-month intervention. They were requested to maintain a physical activity diary and submit it at their six-month assessment. Each of the three evaluations was conducted by the same trained and experienced researchers, who were blinded to the patient group assignment, to ensure consistency and accuracy. The study is a component of a substantial European multi-center randomized controlled trial that has been registered on ClinicalTrials.gov (NCT05234671). The Aristotle University of Thessaloniki, Greece, Research Ethics Committee reviewed and approved the protocol (No: 176497/2021).

### 2.4. 24 h Electrocardiographic Monitoring and Cardiac Autonomic Function Assessment

The SEER 1000 recorders (GE Healthcare, Chalfont St Giles, UK) were employed to evaluate the cardiac autonomic nervous system activity from the 24 h ambulatory three-channel electrocardiogram (ECG) recordings. HRV and HRT indices were obtained using the CardioDay software (v2.6, GE Healthcare, Chalfont St Giles, UK). Both time-domain and frequency-domain indices were assessed to quantify HRV. The root mean square of successive RR interval differences (rMSSD) and the percentage of successive RR intervals that differ by more than 50 ms (pNN50), both of which show activity in the parasympathetic nervous system, the standard deviation of NN intervals (SDNN), which represents the overall HRV, and the standard deviation of the average NN intervals for each 5 min segment of a 24 h HRV recording (SDANN), which represent long-term changes in HRV, were obtained concerning time-domain indices. The indices that were analyzed in the frequency domain were as follows: the very-low-frequency (VLF) band (0.0033–0.04 Hz), which reflects long-term autonomic influences, the low-frequency (LF) band (0.04–0.15 Hz), which reflects both sympathetic and parasympathetic influences, the high-frequency (HF) band (0.15–0.4 Hz), an index of parasympathetic nervous system activity, the total power (TP) reflecting overall HRV, and the ratio of LF-to-HF power (LF/HF), which indicates sympathovagal balance [26]. LF and HF components were evaluated in terms of their absolute (measured in ms^2^) and relative (measured in n.u.) power. In the context of HRT, the turbulence onset (TO) and the turbulence slope (TS) are key parameters. The former indicates the heart rate (HR) changes after a ventricular premature beat, while the latter indicates the deceleration of the HR that follows the acceleration. Normal TO values are less than 0% and TS values are above 2.5 ms/R-R intervals [27].

### 2.5. Arm Crank CPET

Maximal cardiopulmonary exercise tests were conducted on all patients using an arm ergometer (Monark 881E Rehab Trainer, MONARK EXERCISE AB, Vansbro, Sweden), since the exercise training program focused on the upper body. The peak oxygen consumption (VO_2_peak) was evaluated using the Med Graphics Breeze Suite CPX Ultima ergospirometer (Medical Graphics Corp, Saint Paul, MN, USA). The exercise protocol included 3 min warm-up and cool-down periods. In between, a graduated exercise load was applied according to gender. In males, the exercise started with 30 watts and continued with a 10-watt per minute increase, while the females started with 20 watts and continued with a 6-watt per minute increase, until exhaustion. There was continuous electrocardiographic and hemodynamic monitoring during testing. The peak power output (PPO) in watts was assessed for each patient.

### 2.6. Exercise Training Program

After randomization, group ET participated in an individualized mixed-type exercise program, twice a week for three months. Τhe training program was customized to focus on the upper body, as a previous study conducted by our group demonstrated that the endothelial-dependent function enhanced by HIIT upper-limb exercise in comparison to lower-limb exercise [28]. Through the constant supervision of an experienced exercise trainer, each exercise training program started with the aerobic part, which comprised high-intensity interval training for 30 min. The Monark 881E Rehab Trainer was utilized by each patient to complete a five-minute warm-up featuring light arm cranking, followed by 30 s of 100% PPO, 30 s of passive recovery, and a five-minute cooldown. Thereafter, they performed upper-body circuit resistance training with 5 exercises for 15 min. Dumbbells were used in the resistance program, which included shoulder lateral raises, bench press exercises, biceps curls and triceps extensions while seated. They completed 10 repetitions of each exercise at 75–80% of one repetition maximum for three circuits with a recovery period of 2–3 min. Blood pressure (BP), HR, and Borg rate of perceived exertion were evaluated during each session. Throughout the training sessions, the exercise load was gradually adjusted to accommodate individual adaptations in accordance with Borg rate of exertion. Patients were asked to be active for the rest of the days and repeat the exercises by themselves at home, when convenient, for 3 months.

### 2.7. Statistical Analysis

The data are presented as mean ± SD. To investigate the variables’ normal distribution, the Shapiro–Wilk test was implemented. The *t*-test was employed to estimate the between-group differences at baseline and the Χ^2^ for the categorical variables. To compare the mean differences within time and between groups, the two-way ANOVA for repeated measures in conjunction with the Bonferroni post hoc test was employed. To quantify the differences between values at 3 and 6 months “Delta” (Δ) changes were calculated in all variables. To examine the relationships between CPET, HRV, and HRT variables, linear regression analysis was conducted. To ascertain the variables that influence HRV indices and VO_2_peak, multiple regression analysis was implemented. The statistical analyses were conducted using IBM SPSS Statistics 29.0 for Windows (Chicago, IL, USA).

## 3. Results

**Figure 1:**
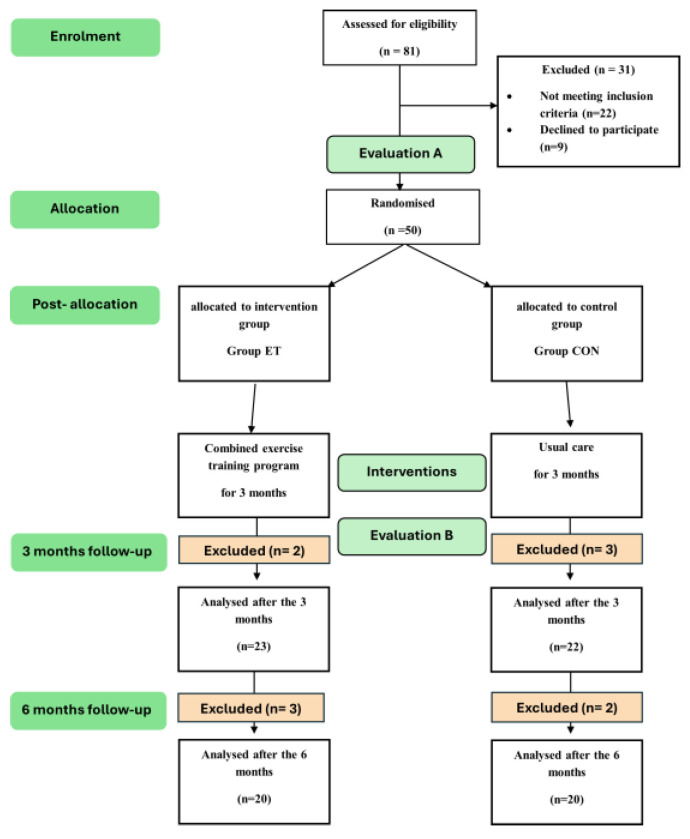
Diagram of the participants’ flow.

Out of the 81 patients with SSc who were evaluated for eligibility, 22 patients did not meet the inclusion criteria and nine declined to participate. As a result, 50 patients were randomly assigned to one of the two groups. The analysis was conducted on the 20 patients from each group who remained at the conclusion of the six-month study. Figure 1 illustrates the participants’ flow chart.

Table 1 displays the clinical indicators of the patients. There were no statistically significant differences between the two groups in any of the variables examined at the outset of the study (Table 2 and Table 3). After three months, the ET group exhibited a significant increase in VO_2_peak (mL/kg/min) by 20.82% (from 14.07 ± 4.9 to 17.0 ± 5.5, *p* = 0.001), SDNN by 15.45% (*p* = 0.002), SDANN by 11.47% (*p* = 0.04), rMSSD by 32.6% (*p* = 0.017), pNN50 by 57.14% (*p* = 0.01), HF (ms^2^) by 20.14% (*p* =0.01), HF (n.u.) by 15.23% (*p* = 0.01) and TS by 25.4% (*p* = 0.01). Conversely, the LF (n.u.) and LF/HF decreased by 14.71% (*p* = 0.01) and 12.03% (*p* = 0.004). All exercised patients completed 24 sessions of exercise, twice a week. There was no exercise-related adverse effect in any patient of the ET group.

The exercise group experienced a decrease in VO_2_peak (mL/kg/min) by 18.82% (from 17.0 ± 5.5 to 13.8 ± 6.4, *p* = 0.003), in HRmax (bpm) by 2.66% (*p* = 0.01), in HRmax (%) by 5.0% (*p* = 0.01), in SDNN by 11.72% (*p* = 0.01), in SDANN by 7.8% (*p* = 0.05), in rMSSD by 20.11% (*p* = 0.01), in pNN50 by 26.13% (*p* = 0.009), in HF (ms2) by 10.7% (*p* = 0.04), in HF (n.u.) by 11.91% (*p* = 0.005), in TS by 10.8% (*p* = 0.03), and in HR at rest by 6.17% (*p* = 0.04) between three and six months. No statistically significant difference was observed in the group ET between baseline and six months.

Following three months, there was no statistically significant difference in the VO_2_ peak or any HRV and HRT index in the group CON. Conversely, there was a statistically significant decrease in VO_2_peak, as measured in L/min and mL/kg/min by 19.8% (from 0.91 ± 0.2 to 0.73 ± 0.1, *p* = 0.001) and 18.9% (from 13.2 ± 3.4 to 10.7 ± 2.5, *p* = 0.001), respectively, as well as in HRmax, as measured in bpm and % by 10.6% (*p* = 0.001) and 12.22% (*p* = 0.001), respectively, and pNN50 by 29.03% (*p* = 0.03) after six months in the group CON. There was a statistically significant difference in all time-domain variables, as well as HF, TS, VO_2_peak, and HR max, when the two groups were compared at three months. In the group ET, the HF, as expressed in both absolute power (ms^2^ and normalized units), increased by 22.4 (*p* < 0.001) and 27.3% (*p* < 0.001), respectively, and TS by 20.2% (*p* = 0.004). When comparing the two groups at three months the results in Table 3 show that VO_2_peak, as expressed in absolute terms (L/min) and relative terms (mL/kg/min), increased by 50.6% (1.62 ± 1.8 in ET compared to 0.80 ± 0.1 in CON, *p* = 0.05) and 30.5% (17.0 ± 5.5 in ET compared to 11.8 ± 1.4 in CON, *p* = 0.001), respectively, and HRmax, as expressed in bpm and the percentage of the maximum, increased by 11% (127.4 ± 22.4 in ET compared to 113.4 ±10.1, *p* = 0.01) and 10% (76.8 ± 13.4 in ET compared to 69.1 ± 7.77 in CON, *p* = 0.02), respectively, in comparison to the group CON. During the detraining period, only 30% of the ET patients (6 out of 20 patients) continued to be physically active. However, the control group displayed deterioration, whereas the ET group did not exhibit a statistically significant difference from the baseline values at six months.

**Figure 2:**
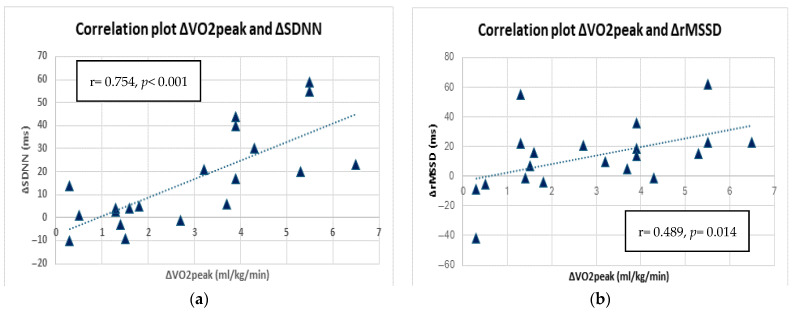
Correlation plot between ΔVO_2_peak and (**a**) ΔSDNN and (**b**) ΔrMSSD in group ET at 3 months.

A linear regression analysis was conducted to determine whether the increase in peak oxygen uptake (ΔVO_2_peak) was correlated with changes in cardiac autonomic function (ΔHRV and HRT parameters) within group ET. A robust positive correlation was observed between ΔVO_2_peak and ΔSDNN (r = 0.754, *p* < 0.001), as indicated by the analysis (Figure 2a). Moreover, moderate positive relationship was observed between VO_2_peak and ΔrMSSD (r = 0.489, *p* = 0.014), which was statistically significant (Figure 2b).

To determine which indices predict the sympathovagal balance between the baseline and the three months follow up for all participants, a multiple regression analysis was conducted, with ΔSDNN serving as a subordinate variable (Table 4). The independent variables include ΔVO_2_peak (mL/kg/min), ΔLF/HF ratio, SCL70 antibodies and group allocation. The model accounted for 49,7 percent of the variability observed in ΔSDNN (R^2^ = 0.497, F = 8.648, *p* < 0.001), SCL70 (*p* = 0.041) and change in VO_2_peak (*p* < 0.001) were identified as significant contributors to ΔSDNN, indicating a potential modulatory effect of SCL70 antibodies on autonomic adaptations and that greater improvements in aerobic capacity were associated with higher increases in SDNN.

Multiple regression analysis was also performed to examine the extent to which group allocation, disease duration, changes in SDNN, and LF/HF ratio, predicted variation in VO_2_peak (ΔVO_2_peak) across the cohort over the 3 months period (Table 5). A statistically significant model (R^2^ = 0.759, F = 27.588, *p* < 0.001) was identified in the analysis, which accounted for 75.9 percent of the total variance in ΔVO_2_peak, presenting a strong explanatory model. Group allocation (*p* < 0.001), disease duration (*p* = 0.014), and change in SDNN (*p* < 0.001) were regarded as significant contributors to change in VO_2_peak, suggesting that longer disease chronicity is associated with diminished gains in aerobic capacity and enhancements in autonomic regulation are linked to improvements in cardiorespiratory efficiency.

## 4. Discussion

The findings of this study suggest that a three-month mixed-type exercise training intervention can enhance cardiac autonomic function and cardiorespiratory efficiency, as well as significantly delay the progression of deterioration in SSc patients over time. Another important finding of this study is that cardiorespiratory efficiency contributes to cardiac autonomic function and vice versa, highlighting the importance of an active lifestyle and exercise interventions in the SSc population.

Our SSc patients showed sympathetic predominance, reduced vagal modulation and sympatho-vagal dysfunction worsened over time, as observed in our control group during the six months. There is a known relationship between cardiac autonomic dysfunction and SSc, characterized by reduced parasympathetic activity and sympathovagal imbalance. This relationship is established in the early stages of the disease and continues to develop over time [16]. The increased sympathetic and reduced vagal-mediated HRV in SSc patients was found to be correlated with lower sleep and quality of life scores, as well as a higher incidence of arrhythmia [10,29]. Several possible pathogenetic mechanisms are attributed to cardiac autonomic dysfunction, such as neurotrophin receptors blockage, inflammation, hypoxia, and ischemia. Especially sympathetic nervous system is found to be more sensitive to ischemia, leading to an excessive increase in catecholamine levels, which is also connected to the vascular derangement observed in SSc [16].

The 3-month exercise training intervention led to an increase in all HRV indices reflecting vagal activity and sympathovagal balance. No prior research has investigated the impact of exercise training on cardiac autonomic function in patients with SSc. However, research conducted on patients with chronic diseases, such as diabetes mellitus, chronic kidney disease and cancer has demonstrated that exercise training is a viable intervention for cardiac autonomic dysfunction [18,19,21,22]. A two-year physical activity program was found to restore cardiac autonomic function and specifically parasympathetic tone, as measured by heart rate recovery, in patients with rheumatoid arthritis [30]. This is crucial because cardiac autonomic dysfunction is a recognized factor in an elevated risk of cardiovascular disease, sudden cardiac death and a worse prognosis [31]. It is already known that high-intensity exercise promotes increased acute stress, affecting respiratory, cardiovascular, and metabolic parameters and resistance exercise training affects the baroreflex-NO axis [32]. There is evidence that the combination of strength and aerobic exercise enhances the HRV results of aerobic alone exercise training [33,34]. We found a strong relationship between the improvement of parasympathetic activity, as expressed by rMSSD, and sympathovagal balance, as expressed by SDNN and the improvement in cardiorespiratory efficiency in our exercised patients. In addition, the sympathovagal balance was significantly influenced by peak oxygen uptake levels and participation in the exercise intervention, as indicated by multiple regression analysis.

Significant enhancements in cardiorespiratory efficiency were observed after the three-month mixed-type exercise training intervention. The HRV indices that reflect parasympathetic activity and sympathovagal balance were significant contributors to the peak oxygen uptake levels. In a previous study of our research group, the same mixed-type exercise protocol caused improved endothelial-dependent reactivity, indicating an improvement in microvascular function in SSc patients [35]. Exercise-induced adaptations in cardiorespiratory efficiency and cardiac autonomic function in our SSc patients may be explained by microvascular adaptations to exercise training. In addition, we observed that the systolic function of the right ventricle was enhanced in SSc patients with subclinical RV dysfunction following three months of exercise training [36]. The clinical outcomes of this enhancement in VO_2_peak may have been favorable, as an increase in VO_2_peak was associated with a lower rate of hospital readmission in patients with coronary artery disease who were undergoing secondary prevention [37].

After three months of follow-up, all favorable effects of exercise were lost and the examined indices returned to baseline values. However, this gives added value to our exercise intervention, since there was deterioration over time in the control group. Exercise training managed to prevent this deterioration in our exercise SSc patients, indicating that there is a need for a continuous active lifestyle.

Τhe study’s results indicate that a three-month mixed-type exercise training intervention can significantly impede the progression of deterioration in SSc patients and improve cardiac autonomic function and cardiorespiratory efficiency. It is imperative to consider the positive impact of exercise training on cardiac autonomic function, as autonomic dysfunction is a primary cause of morbidity and mortality in SSc. Another significant finding is the bidirectional relationship between cardiac autonomic function and cardiorespiratory efficiency, which underscores the significance of structured exercise interventions and an active lifestyle in this population. The findings should be seen considering some limitations. Firstly, the sample size is relatively small, and secondly, we have not examined the long-term follow-up effects. Moreover, the cardiac autonomic function results are not connected with clinical outcomes and prognosis. Future studies could investigate the underlying mechanisms that connect cardiorespiratory efficiency and autonomic regulation, compare various exercise modalities or intensities and examine the long-term effects of exercise training on cardiac autonomic function in SSc.

## 5. Conclusions

The findings of this study suggest that a three-month mixed-type exercise training program results in a substantial increase in parasympathetic activity, a decrease in sympathetic activity, and an enhancement in the balance between the two branches in the SSc patients. To mitigate the gradual decline in cardiorespiratory capacity and cardiac autonomic function, it is advised to maintain an active lifestyle. These findings validate the incorporation of supervised exercise programs into standard care for SSc from a practical perspective. They provide a non-pharmacological approach to improve clinical outcomes and potentially reduce the healthcare burden over time.

## Figures and Tables

**Table 1:** Clinical parameters of the SSc patients at the start of the study (data are shown as mean ± SD).

Characteristics	Total Mean ± SD	Group ET Mean ± SD	Group CON Mean ± SD	*p*-Value
N	40	20	20	-
Age (years)	57.6 ± 10.8	55.9 ± 9.0	59.3 ± 12.4	0.320
Gender				
Male (n, %)	6 (15.0%)	1 (5.0%)	5 (25.0%)	0.182
Female (n, %)	34 (85.0%)	19 (95.0%)	15 (75.0%)	
Body Mass Index (kg/m^2^)	27.5 ± 4.3	27.1 ± 4.6	27.8 ± 4.0	0.604
Disease duration (years)	8.7 ± 5.3	7.7 ± 5.4	9.8 ± 5.2	0.230
Type of disease				
Limited (n, %)	23 (57.5%)	12 (60.0%)	11 (55.0%)	0.749
Diffuse (n, %)	17 (42.5%)	8 (40.0%)	9 (45.0%)	
ANA				
Positive (n, %)	19 (95.0%)	19 (95.0%)	19 (95.0%)	1.000
Negative (n, %)	2 (5.0%)	1 (5.0%)	1 (5.0%)	
ACA				
Positive (n, %)	30 (75.0%)	15 (75.0%)	15 (75.0%)	1.000
Negative (n, %)	10 (25.0%)	5 (25.0%)	5 (25.0%)	
SCL70				
Positive (n, %)	19 (47.5%)	8 (40.0%)	11 (55.0%)	0.342
Negative (n, %)	21 (52.5%)	12 (60.0%)	9 (45.0%)	
CRP (mg/L)	2.6 ± 6.6	2.1 ± 2.1	2.9 ± 9.2	0.698

ANA: antinuclear antibodies; ACA: anti-centromere antibodies; SCL70: serum anti-topoisomerase I antibody; CRP: C-reactive protein; significant at the 0.05 level (*p* < 0.05).

**Table 2:** Heart rate variability and heart rate turbulence parameters in both groups at the start of the study and after 3 and 6 months (Data are presented as mean ± SD).

		Group ET			Group CON		ET vs. CON		
	Baseline	After 3 Months	After 6 Months	Baseline	After 3 Months	After 6 Months	Baseline *p*-Value	After 3 Months *p*-Value	After 6 Months *p*-Value
**HRV**									
HR (bpm)	77.6 ± 9.5	72.9 ± 6.1	77.4 ± 6.7 ^b^	77.0 ± 6.0	78.2 ± 5.8	76.2 ± 5.8	*p* = 0.83	* *p* = 0.02	*p* = 0.55
TP (ms^2^)	8330.3 ± 2038.9	9230.3 ± 1099.5	8721.5 ± 1896.7	8591.0 ± 1916.0	8636.0 ± 1929.7	8246.3 ± 1849.1	*p* = 0.64	*p* = 0.26	*p* = 0.43
Mean 24-RR intervals(ms)	816.2 ± 73.8	808.6 ± 58.0	811.2 ± 74.2	812.2 ± 67.0	818.6 ± 66.4	817.1 ± 56.4	*p* = 0.85	*p* = 0.50	*p* = 0.78
**HRV Time domain variables**									
SDNN (ms)	104.2 ± 11.8	120.3 ± 15.6 ^a^	106.2 ± 16.8 ^b^	103.9 ± 12.6	102.0 ± 25.7	97.4 ± 19.5	*p* = 0.93	* *p* = 0.002	*p* = 0.14
SDANN (ms)	90.6 ± 15.2	101.0 ± 13.7 ^a^	93.1 ± 16.8 ^b^	85.7 ± 10.0	83.4 ± 26.4	83.5 ± 28.4	*p* = 0.28	* *p* = 0.006	*p* = 0.20
rMSSD (ms)	40.5 ± 17.9	53.7 ± 16.3 ^a^	42.9 ± 11.9 ^b^	42.7 ± 18.4	38.5 ± 9.4	34.5 ± 7.1	*p* = 0.69	* *p* = 0.002	*p* = 0.74
pNN50 (%)	5.6 ± 5.2	8.8 ± 2.0 ^a^	6.5 ± 3.1 ^b^	6.2 ± 3.2	5.2 ± 1.7	4.4 ± 1.7	*p* = 0.63	* *p* < 0.001	*p* = 0.84
**HRV Frequency domain variables**									
VLF (ms^2^)	2670.5 ± 1612.2	2694.4 ± 1073.1	2751.4 ± 677.7	2721.0 ± 1049.7	2849.9 ± 936.9	2672.4 ± 1060.4	*p* = 0.89	*p* = 0.63	*p* = 0.78
LF (ms^2^)	2832.5 ± 648	2686.1 ± 719.4	2861.2 ± 1094.3	2735.6 ± 414.0	3097.0 ± 1460.1	3149.3 ± 1034.1	*p* = 0.57	*p* = 0.28	*p* = 0.60
HF (ms^2^)	2590.6 ± 677.2	3112.5 ± 295.9 ^a^	2778.3 ± 649.8 ^b^	2551.7 ± 862	2415.4 ± 544.4	2409.9 ± 793.6	*p* = 0.84	* *p* < 0.001	*p* = 0.25
LF (n.u.)	50.3 ± 6.8	42.9 ± 9.0 ^a^	47.4 ± 10.3	49.0 ± 11.8	51.2 ± 13.9	54.4 ± 16.6	*p* = 0.65	*p* = 0.06	*p* = 0.12
HF (n.u.)	46.6 ± 8.4	53.7 ± 6.3 ^a^	47.3 ± 8.6 ^b^	45.7 ± 13.4	39.0 ± 14.5	39.2 ± 11.8	*p* = 0.77	* *p* < 0.001	*p* = 0.79
LF/HF	1.08 ± 0.2	0.95 ± 0.1 ^a^	0.98 ± 0.2	1.05 ± 0.3	1.05 ± 0.4	1.01 ± 0.4	*p* = 0.76	*p* = 0.39	*p* = 0.09
**Heart rate turbulence**									
TO (%)	−0.006 ± 0.02	−0.004 ± 0.01	−0.008 ± 0.09	0.0021 ± 0.02	−0.0032 ± 0.00	−0.004 ± 0.00	*p* = 0.30	*p* = 0.68	*p* = 0.15
TS (ms/RR)	5.9 ± 2.5	7.4 ± 1.4 ^a^	6.6 ± 1.5 ^b^	6.1 ± 2.4	5.9 ± 1.7	5.0 ± 1.4	*p* = 0.87	* *p* = 0.004	* *p* < 0.001
Mean RR intervals (ms)	775.2 ± 86.5	774.3 ± 85.7	753.6 ± 91.5	768.8 ± 111.6	783.3 ± 83.3	766.7 ± 75.2	*p* = 0.83	*p* = 0.79	*p* = 0.63

HRV: heart rate variability; HR: heart rate; TP: total power; RR intervals: time intervals between two successive heartbeats; SDNN: standard deviation of RR intervals; SDANN: standard deviation of the average NN intervals for each 5 min segment of a 24 h HRV recording; rMSSD: root mean square of successive RR interval differences; pNN50: the number of pairs of successive NN (R-R) intervals that differ by more than 50 ms; VLF: very low frequency; LF: low frequency; HF: high frequency; TO: turbulence onset; TS: turbulence slope; significant at the 0.05 level (*p* < 0.05); ^a^ *p*-value differences between baseline and 3 months in the ET group; ^b^ *p*-value differences between 3 months and 6 months in the ET group; *: *p* < 0.05 (significant).

**Table 3:** Cardiorespiratory variables in both groups at the start of the study and after 3 and 6 months (Data are presented as mean ± SD).

		Group ET		Group CON				ET vs. CON	
	Baseline	After 3 Months	After 6 Months	Baseline	After 3 Months	After 6 Months	Baseline *p*-Value	After 3 Months *p*-Value	After 6 Months *p*-Value
VO_2_peak (L/min)	1.01 ± 0.4	1.62 ± 1.8	1.01 ± 0.4	0.91 ± 0.2	0.80 ± 0.1	0.73 ± 0.1	*p* = 0.34	* *p* = 0.05	* *p* = 0.02
VO_2_peak (mL/kg/min)	14.07 ± 4.9	17.0 ± 5.5 ^a^	13.8 ± 6.4 ^b^	13.2 ± 3.4	11.8 ± 1.4	10.7 ± 2.5	*p* = 0.47	* *p* = 0.001	*p* = 0.056
HRmax (bpm)	128.3 ± 22.8	127.4 ± 22.4	124.0 ± 23.4 ^b^	120.2 ± 21.1	113.4 ± 10.1	107.4 ± 20	*p* = 0.19	* *p* = 0.01	* *p* = 0.02
HRmax (%)	76.7 ± 15.8	76.8 ± 13.4	72.9 ± 14 ^b^	72.8 ± 13.5	69.1 ± 7.77	63.9 ± 13.0	*p* = 0.43	* *p* = 0.02	* *p* = 0.04

VO_2_peak: peak oxygen uptake; HRmax: maximum heart rate; significant at the 0.05 level (*p* < 0.05). ^a^ *p*-value differences between baseline and 3 months in the ET group; ^b^ *p*-value differences between 3 months and 6 months in the ET group; *: *p* < 0.05 (significant).

**Table 4:** ΔSDNN was used as the dependent variable in multiple regression analysis between baseline and 3-month follow-up for both groups.

Model	B	Beta	t	*p*-Value
Constant	−20.628	-	−1.417	0.165
Group	12.082	0.282	1.418	0.165
SCL70	11.604	0.271	2.125	0.041 *
ΔVO_2_peak (mL/kg/min)	5.591	0.795	3.870	<0.001 *
ΔLF/HF	5.132	0.149	1.123	0.269
R^2^= 0.497 F = 8.648				

ΔVO_2_peak: peak oxygen uptake difference; ΔLF/HF: low frequency (LF) to high frequency (HF) difference; *: *p* < 0.05 (significant).

**Table 5:** ΔVO_2_peak was used as the dependent variable in multiple regression analysis between baseline and 3-month follow-up for both groups.

Model	B	Beta	t	*p*-Value
Constant	6.730	-	6.712	<0.001 *
Group	−3.580	−0.589	−6.298	<0.001 *
Disease duration (years)	−0.131	−0.228	−2.598	0.014 *
ΔSDNN	−0.050	0.349	3.605	<0.001 *
ΔLF/HF	−0.047	−0.010	−0.101	0.920
R^2^ = 0.759 F = 27.588				

ΔSDNN: standard deviation of RR intervals (SDNN) difference; ΔLF/HF: low frequency (LF) to high frequency (HF) difference; *: *p* < 0.05 (significant).

## Data Availability

Data can be provided upon request.

## Abbreviations

ANA	Antinuclear antibodies
ACA	Anti-centromere antibodies
CON	Control Group
CRP	C-reactive protein
ET	Exercise training group
HF	High frequency
HR	Heart Rate
HRT	Heart rate turbulence
HRV	Heart rate variability
LF	Low frequency
pNN50	The number of pairs of successive NN (R-R) intervals that differ by more than 50 ms
rMMSD	Root mean square of successive RR interval differences
RR	Time intervals between two successive heartbeats
SDANN	Standard deviation of the average NN intervals for each 5 min segment of a 24 h
SDNN	Standard deviation of RR intervals
TO	Turbulence onset
TP	Total Power
TS	Turbulence slope
SCL70	Serum anti-topoisomerase I antibody
SSc	Systemic Sclerosis
VLF	Very low frequency
VO_2_peak	Peak oxygen uptake
